# Biomolecular assemblies through weak noncovalent interactions: Higher-order transient structures and their condensate phase

**DOI:** 10.1073/pnas.2531431123

**Published:** 2026-07-02

**Authors:** Roderick MacKinnon, Christoph A. Haselwandter

**Affiliations:** ^a^ Laboratory of Molecular Neurobiology and Biophysics, HHMI, The Rockefeller University, New York, NY 10065; ^b^ Department of Physics and Astronomy, University of Southern California, Los Angeles, CA 90089; ^c^ Department of Quantitative and Computational Biology, University of Southern California, Los Angeles, CA 90089

**Keywords:** higher-order transient structures, HOTS, biomolecular condensates, membrane signaling, molecular crowding

## Abstract

Recent data suggest that many membrane proteins spontaneously organize into spatial patterns through weak noncovalent interactions. These weak interactions are protein type-specific and underlie the formation of higher-order transient structures (HOTS), which can function as 10 to 100 nanometer-sized, transient hubs of membrane signaling. We describe the necessary conditions for HOTS assembly to occur, its thermodynamic relationship to biomolecular condensate formation, and potential roles of HOTS in biology stemming from their unique physical properties. Currently, a quantitative understanding of HOTS is limited to membrane proteins, but many observations suggest that HOTS may also be abundant in three-dimensional cellular compartments.

Because even small cells are orders of magnitude larger than covalently bonded biomolecules, life depends on a multitude of noncovalent chemical interactions to build cells and carry out their biochemical processes. Noncovalently assembled structures are abundant in many recognizable forms—as multisubunit protein “machines,” viral capsids, lipid membranes, and many more (1). These familiar assemblies are generally characterized by relatively strong noncovalent interactions (many *k_B_T*) such that the equilibrium between the covalent building blocks (the subunits or protomers) and the assembled structures is shifted far toward the latter. The stability and relative permanence of this kind of noncovalent assembly are prerequisites to their biological function.

This perspective focuses on a different kind of noncovalent assembly that we know much less about—the kind mediated by weak (few *k_B_T*) yet specific noncovalent interactions. Biomolecular assemblies formed by weak interactions are transient and often too small to see without specialized methods of detection. Here, we summarize properties of weak noncovalent biomolecular interactions with the aim to highlight three key concepts.

First, the existence of weak noncovalent assemblies is counter-intuitive because they should be diminishingly rare at the very low protomer concentrations frequently found in cells, and yet they do exist because of certain physical properties of the molecular environment in living cells.

Second, and central to this perspective, weak noncovalent interactions create two distinct forms of biochemical matter. The first form, which we named higher-order transient structures (HOTS), exists as a distribution of small (in protomer number) oligomers characterized by a monotonically decreasing size distribution (2). This is the form referenced above as difficult to see using conventional light microscopic methods because their size is typically less than the Abbe diffraction limit. The second form is a condensate, which is larger and easier to see (3, 4). As we will explain, HOTS and condensates can coexist and be created by the same physical interactions and thermodynamic driving forces; however, they are distinct forms of assembly, have different physical properties, and probably fulfill different roles in biology.

Third, weak interactions together with their small average protomer number render HOTS intrinsically unstable and transient. Transience enables HOTS to fulfill biological functions that depend on rapid assembly and disassembly of oligomeric structures.

To place assemblies mediated by weak noncovalent interactions into the broader context of noncovalent assemblies observed in biology, Fig. 1 exhibits three different categories. In the first, protomers assemble to form structures with a fixed stoichiometry. The term “limited” refers to the property that the assembly does not keep growing but stops at a fixed stoichiometry. The noncovalent interactions that bind the protomers are strong, meaning that at equilibrium few individual protomers or substoichiometric assemblies exist. This category includes the abundance of familiar macromolecular assemblies including motor proteins, viral capsids, transporters, channels, and many others.

**Fig. 1.**
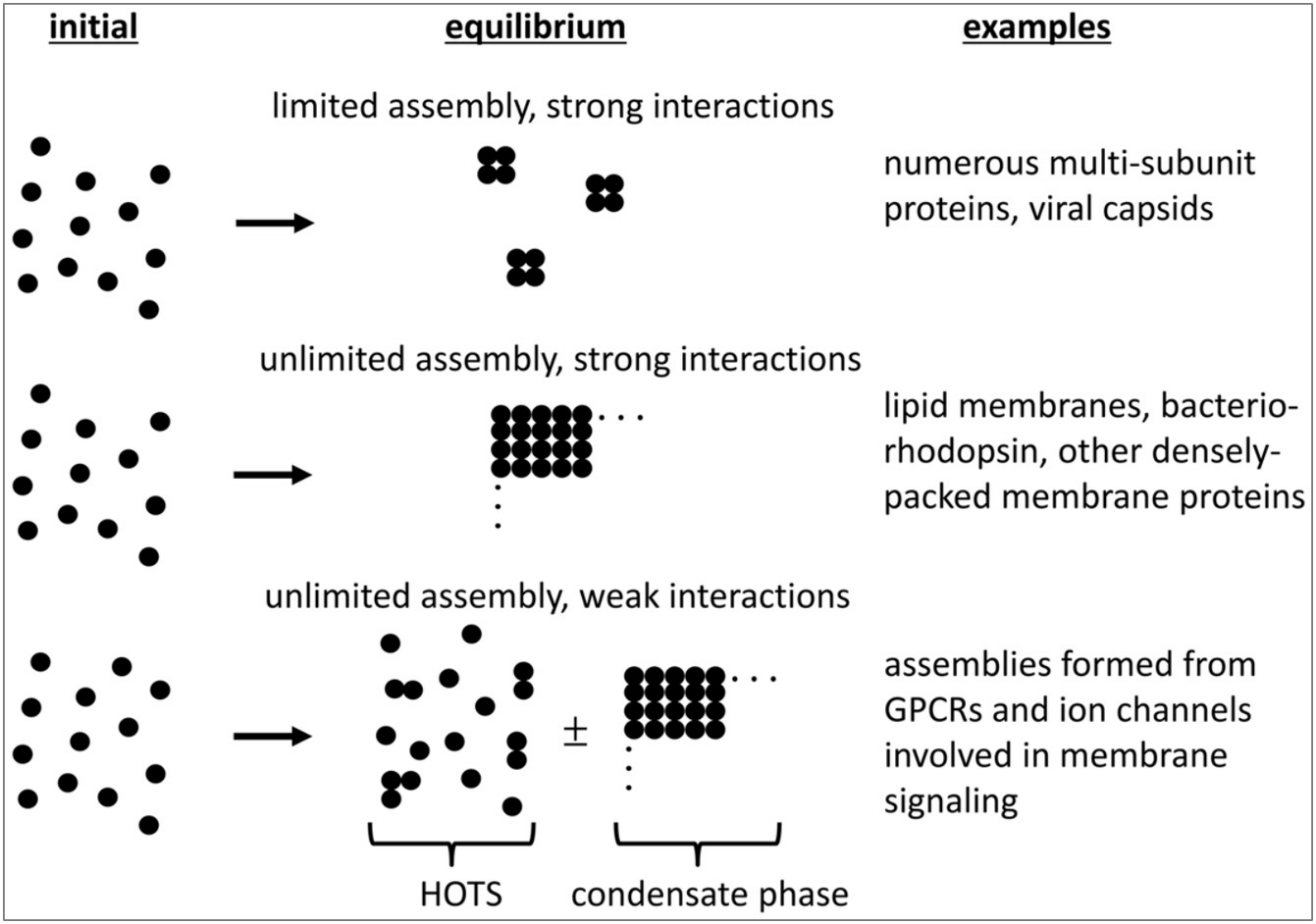
Examples of noncovalent assembly in biology. The first (*Top*) category represents multimeric protein complexes consisting of a fixed stoichiometry of protomers (black disks) that remain strongly associated. The second (*Middle*) category represents protomers that associate strongly to form continuous materials. Bilayer membranes, in which lipid molecules serve as protomers, correspond to this second category in two dimensions (plane of the membrane) and to the first category (a dimer) in the third dimension. The third (*Bottom*) category is the subject of this perspective. Protomers, which can be individual molecules or collections of molecules, interact weakly (a few *k*_*B*_*T*) through multiple interaction sites. A distribution of small oligomers called HOTS forms through these weak but specific interactions. If a saturating concentration of protomers is reached, a first-order phase transition results in a condensate phase, which is in equilibrium with the saturated HOTS distribution.

In the second category in Fig. 1, protomers also bind strongly but assemble without a fixed stoichiometry, thus the designation “unlimited” growth (5). Again, because the noncovalent interactions are strong, at equilibrium few individual protomers or small assemblies occur. Lipid molecules forming lipid bilayers exemplify this category, with (approximately) unlimited growth in the plane of the membrane. The rarity of individual lipid molecules in the aqueous solution reflects the strong noncovalent interactions that bind lipid molecules to reside within the lipid bilayer.

The third category in Fig. 1 and focus of this perspective, which we refer to as “weak, unlimited assembly,” is like the second except that the interactions between protomers are weak. Weak binding means that a measurable fraction of the protomers exist as a distribution of small oligomers, HOTS, which can be in equilibrium with the unlimited assembly (labeled as a condensate phase). As we explain, below, whether the condensate phase is present along with the HOTS depends on the total concentration and bonding properties of the assembling protomers (2). Thus, two distinct forms of biochemical matter, HOTS and a condensate phase, must be considered in this category of weakly interacting components. To be clear, monomers and substoichiometric aggregates also exist, in principle, in the first and second categories of noncovalent assembly, but their concentrations are so small that they are functionally unimportant. By contrast, in the third category, the interactions yielding biomolecular assemblies are weak, and the resulting HOTS are therefore sufficiently abundant to function in biology.

Across the full range of noncovalent assemblies in biology, it is likely that the strength of noncovalent interactions varies continuously from strong to weak. It is also possible that the distinction between limited and unlimited growth is blurred in some biological oligomers in which certain oligomeric states might exist in excess but not to the exclusion of others (6). Thus, Fig. 1 shows three categories taken from what is likely a continuum along axes of growth (from limited to unlimited) and strength of interaction (from strong to weak). Our focus is on the third category of weak, unlimited assembly.

## Concept 1: Molecular Crowding Permits Weakly Interacting Protomers to Assemble At Low Concentrations

If protomers associate only weakly to form oligomers, then dissociated states should be favored. Thus, one would naturally think that oligomers should only be observed when the concentration of protomers is high enough to favor formation of some oligomers by mass action. However, paradoxically, HOTS are observed at very low protomer concentrations. In plasma membranes, for instance, HOTS can form at concentrations of only a few protomers per μm^2^ (2). This can be explained if one considers the cellular environment of the oligomerization reaction (7). For example, many cellular compartments, including regions of cell membranes, are crowded with macromolecules of many kinds (8–12). Most straightforwardly, a crowded environment may reduce the number of configurational microstates adoptable by protomers and oligomers. As a result, the cellular environment may effectively decrease the configurational entropy contribution to the free energy of oligomerization, favoring oligomerization even when the protomer interactions are weak and their concentrations low (7). More generally, macromolecular crowding is expected to yield both enthalpic and entropic contributions to the free energy of HOTS formation (8–12).

The physical environment provided by cellular compartments thus likely plays an important role in the formation of weak noncovalent assemblies (7–12). In cell membranes where HOTS have been characterized quantitatively, it appears that especially crowded regions of the membrane favor HOTS formation (2). And similar proteins can oligomerize to different extents in different cells, apparently because the physical environments are different—for example, in the degree of membrane crowding. These observations underscore the idea that cells might regulate HOTS formation by creating specialized, crowded environments. The important point is, crowded environments in living cells can promote the formation of noncovalent assemblies from weakly interacting components, permitting HOTS to form at low protomer concentrations.

## Concept 2: Weak Interactions Mediate Two Distinct But Related Forms of Self-Assembly, HOTS, and Condensates

**Fig. 2.**
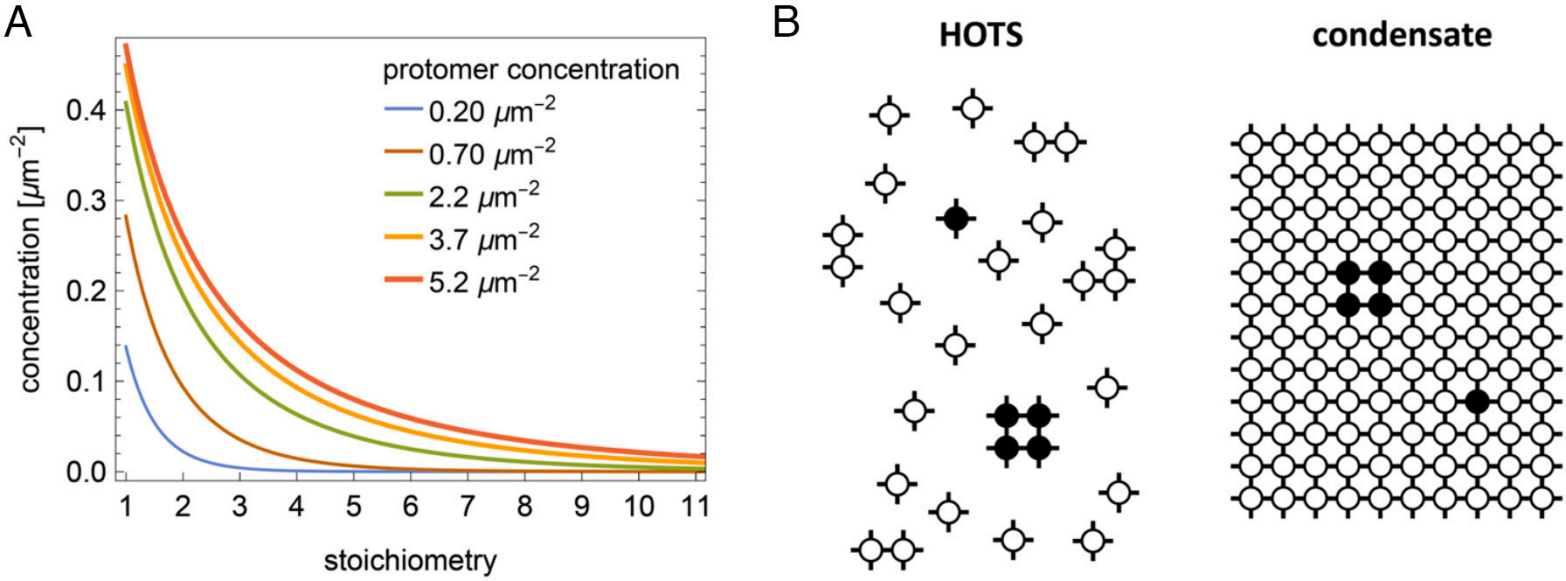
The form of the HOTS size distribution and why it can give rise to a condensate phase. (*A*) Generic graph of the concentration of monomers, dimers, etc. for distinct total protomer concentrations (legend) up to the saturating concentration, illustrating the monotonically decreasing shape of the HOTS size distribution and its broadening with increasing total protomer concentration. Numerical values are chosen based on data on GPCR HOTS in HL-1 plasma membranes (2, 7). (*B*) Schematic of monomers, dimers, trimers, and tetramers of protomers “in solution” (*Left* panel). Each protomer can make multiple (in this example, four) bonds with neighboring protomers. One monomer and one tetramer are marked in black to illustrate that a monomer contains four bonds on its perimeter whereas a tetramer contains eight bonds on its perimeter. The *Right* panel depicts an idealized condensate, in which we highlight an absorbed monomer and an absorbed tetramer. For illustrative purposes, the structures of the HOTS and of the condensate are shown as idealized, regular lattices, but this is not a requirement. Each bond represents a cohesive, reversible point of contact. Bonds are generally noncovalent, can be formed from ordered or intrinsically disordered protein segments, can be mediated by smaller molecules or ions, and need not be identical. A protomer could be a single macromolecule, for example a protein, or a complex of different macromolecules.

A HOTS distribution is the equilibrium distribution of small oligomers formed reversibly through weak, unlimited assembly. Thus, HOTS do not have a fixed stoichiometry. Imagine, for example, identical protomers undergoing a random walk in a two-dimensional plane, say, a membrane. If these protomers bind to each other when they come into contact, they would form oligomers. Because their binding is reversible, they would continuously assemble and disassemble, that is, they would be transient. But at any given time, a snapshot of this process would show some concentration of monomers, dimers, trimers, etc. At very low protomer concentrations nearly every protomer would be a monomer because encounters would be rare, but as the protomer concentration is increased, oligomers (HOTS) would appear and the distribution of sizes (i.e., the number of protomers in a HOTS) would broaden, as shown in Fig. 2*A*. By the very same physical mechanism as in two dimensions, a HOTS distribution will occur for reversibly binding protomers in three dimensions, for example in the cytoplasm and in organelles including the nucleus, but the process is easier to depict—and easier to measure experimentally—in two dimensions, for example, in a membrane.

The HOTS distribution exhibits two essential properties, which are both manifestations of the second law of thermodynamics for weak, unlimited assembly: first, it is monotonically decreasing (Fig. 2*A*); and second, except under special conditions (see below) the distribution can only contain up to a certain maximum concentration of protomers. This second property, at first surprising, lies at the heart of understanding the relationship between HOTS and their condensate phase (Fig. 2*B*).

The monotonically decreasing shape of the HOTS distribution (Fig. 2*A*) minimizes the system’s free energy by striking a balance between the configurational entropy contribution (which favors dissociated forms) and the chemical contribution (i.e., cohesiveness, which favors associated forms). The formation of a condensate phase results for the same reason, but the condensate is not a large HOTS. One way to convey this important idea is through appeal to familiarity. Consider a saturated solution of some solute x that is in equilibrium with an undissolved form of the solute, say, a condensate phase. We usually picture the dissolved form of x as single protomers in solution but, given that x can interact with itself to form a condensate, it can in principle also form oligomers in solution through the same interactions that exist in the condensate (Fig. 2*B*). For weak, unlimited assembly, these oligomers are HOTS and will adopt a monotonically decreasing size distribution. HOTS that coexist in equilibrium with their condensate phase comprise the saturated HOTS distribution. While HOTS are small, the condensate can in principle contain an arbitrarily large number of x. As a result, addition or subtraction of x to a saturated system, given sufficient time to equilibrate, will make the condensate grow or shrink, but the HOTS distribution will not change perceptibly. If x is removed from the system to the point that the condensate disappears, further removal of x will cause the HOTS distribution to become smaller and narrower, as shown in Fig. 2*A*.

At the level of chemical interactions, how can we understand why a HOTS distribution stops growing, and a condensate appears at a defined concentration of protomers? Consider the depiction in Fig. 2*B* of monomers and HOTS (together comprising a solution phase) in equilibrium with their condensate phase. Each protomer can form up to four cohesive bonds in this example. While bonding is energetically favorable, the reduction in configurational entropy incurred when protomers coalesce is unfavorable and the equilibrium condition balances these competing effects. If we focus first on monomers, we see that four bonds are made when one of them enters the condensate. This will be reflected by an equilibrium concentration of monomers that balances the energy of making four bonds. By contrast, when the tetramer shown enters the condensate, eight bonds are made. Therefore, a tetramer binds to the condensate with higher affinity (because there are more favorable bonds) and thus a lower equilibrium concentration of tetramers is required to counterbalance the higher affinity. In general, if larger oligomers have more bonds on their edge, causing them to bind the condensate with higher affinity, then their equilibrium concentration will be smaller. This diminishing concentration of larger oligomers ultimately limits the growth of the HOTS distribution to a finite total concentration of protomers. The limit is called the saturated HOTS distribution. Protomers added beyond the limit, once equilibrated, will not go into the HOTS distribution (i.e., the solution phase) but instead enter the condensate phase.

Most but not all protomer interactions can give rise to a condensate. To give an example in which they do not, linear oligomers that grow like beads on a string have only two perimeter bonds (at the ends). Thus, as linear oligomers grow, they will not exhibit higher affinity for a condensate. Consequently, the distribution of one-dimensional oligomers will continue to broaden as protomers are added and no phase transition will occur (5). In general, as long as three or more reversible bonds can form between protomers, the concentration of larger oligomers can be suppressed, the HOTS distribution can have a saturation limit, and condensation can occur. Importantly, the bonds depicted in Fig. 2*B* provide an idealized representation of reversible, cohesive contacts. These contacts need not be identical and, because protomers are generally expected to lack symmetry, will often be irregular, heterogeneous, and show a range of affinities.

What kinds of protomers might form HOTS? In a membrane, the protomers could be a particular type of ion channel or G protein–coupled receptor (GPCR), or a complex of membrane proteins and their associated lipids. In three-dimensional cellular compartments, a protomer could be an enzyme, or a complex of different macromolecules like proteins and RNA. The essential idea is if a protein or other macromolecular complex contains cohesive patches, it can form a distribution of small oligomers called HOTS. Given three or more (not necessarily identical) bonds between protomer units, if enough protomer units are present a first-order phase transition can occur, resulting in a condensate phase in equilibrium with the solution phase comprising a saturated HOTS distribution. The simple picture to have in mind is this: Once the HOTS distribution is saturated, additional protomers will “spill over” into a condensate phase. The composition of and contacts within HOTS and their condensate phase can be identical, but HOTS are small oligomers (Fig. 2*B*), most often smaller than the diffraction limit of visible light, whereas the condensate phase is a continuous, arbitrarily large assembly.

Because HOTS are small (i.e., contain a small number of protomers) they equilibrate rapidly, appearing and disappearing over short timescales measured in seconds. By contrast, the large size of condensates means that they equilibrate over much longer timescales and are more permanent. This important difference between HOTS and their condensate phase raises the question, is one justified in using an equilibrium model to describe HOTS oligomerization and condensation in living cells? After all, in cells protomers are continuously created and recycled, for example inserted into the membrane and then removed, resulting in protomer flows. Furthermore, protomers can become modified over time, for example by binding to a ligand, through phosphorylation or some other chemical modification, which could affect their cohesiveness. There are indeed many processes in cells that keep molecular components out of equilibrium. But if these cellular processes are slow compared to oligomerization, then an equilibrium approximation within a cellular compartment such as the plasma membrane seems appropriate. HOTS, owing to their transience, are likely close to equilibrium. The point of emergence of a condensate phase from a saturated HOTS distribution likewise should follow from an equilibrium or near equilibrium description. But the condensate phase itself, subject to the slow processes of fusion and Ostwald ripening, may be far from equilibrium in a changing cellular environment (13, 14).

The reader by now is likely wondering how the condensates illustrated in Figs. 1 and 2, defined in this perspective as the unlimited assemblies created from saturated HOTS distributions through a first-order phase transition, relate to the biomolecular condensates prevalent in the recent biological literature. Given that the term “biomolecular condensate” is currently used with considerable variability (15, 16), we address this point with trepidation. So far, HOTS have only been characterized quantitatively with membrane proteins because they are arrayed in two dimensions, which facilitates measurement of HOTS size distributions by counting protein labels (2, 17). At sufficiently high protein concentrations, the very same proteins that form HOTS [e.g., the M2 muscarinic GPCR (M2R)] produce macroscopic arrays in membrane reconstitution experiments. These macroscopic arrays have circular shapes and fuse together when they come in contact, i.e., their properties seem to be dominated by a surface energy (18). In other words, the same protein can form HOTS and, at high enough concentrations, can also form two-dimensional (in the membrane) liquid-like condensates that behave like many three-dimensional (inside the cell) condensates described in the literature (3, 4, 19, 20). Furthermore, GPCRs and ion channels, when expressed at low levels and labeled with fluorophores, show individual fluorescent point spread functions (PSFs), and when expressed at higher levels show large clusters up to many microns in diameter, presumably condensates in the membrane that coexist with the PSFs (2). While individual PSFs are not easily distinguishable as monomers or small oligomers, oligomerization can be inferred through PSF merging and splitting events (2, 21–23). Finally, in this perspective we have described the relationship between HOTS and their condensate phase by focusing on the consequence of saturating the HOTS distribution through addition of protomers (or changing the crowded environment in cellular compartments). However, the condensation point is set by the cohesive energy such that stronger (more favorable) interactions will produce condensation at a lower protomer concentration. Consequently, if the protomer interaction energy suddenly increases through, for example, chemical modification of the protomer or some environmental change, a HOTS distribution can abruptly supersaturate, resulting in spontaneous condensation. Thus, the rapid appearance (or disappearance) of condensates on a timescale incompatible with protomer addition in a cellular environment should not be interpreted as incompatibility with HOTS condensation.

Based on the observations outlined in the previous paragraph, we speculate that the first-order phase transition associated with a saturated HOTS distribution could explain biomolecular condensate formation in some cases. If so, an interesting question arises: Which is the functional unit, the condensate or the HOTS? After all, these two forms of self-assembly, while consisting of the same molecules, have different physical properties. Most notably, owing to their size, condensates are relatively long-lived and tend to form at high total protomer concentrations, while HOTS are transient and form not only at but also below the saturating protomer concentration. In the following, we consider attributes of HOTS that might make them uniquely suited for certain biological functions.

## Concept 3: HOTS Can Fulfill Unique Roles in Biology

Many noncovalent biological structures must exhibit stability and relative permanence. Membranes must remain leak-free, viral capsids intact for much of their existence, and multisubunit molecular machines stable and functional. Strong noncovalent interactions underlie these properties. By contrast, other assemblies of biomolecules likely benefit from the transience afforded by weak noncovalent interactions.

Consider, for example, proteins in a signal transduction cascade. Weak noncovalent interactions can produce HOTS of signaling proteins that exist only on the brief timescale of the signaling process. HOTS were described in a cardiac derived cell line in which stimulation of the M2R opens a G protein–gated K^+^ channel (GIRK channel) to slow the rate of action potential occurrence; an outcome that slows heart rate in a native context (2, 17). The problem the M2R HOTS solved in these cells is, a single M2R does not generate a sufficient concentration of G proteins to activate a GIRK channel because the channel’s affinity for the G protein is too low, but a small cluster of M2Rs—in the form of a HOTS—can generate a local G protein concentration that is sufficiently high to activate channels in the vicinity of the M2R HOTS. Moreover, the GIRK channels themselves form HOTS, which apparently through very weak binding interactions between M2R and GIRK channels enforce their proximity, enabling effective control of GIRK channel opening by M2R. The consequence of these weak biomolecular interactions is a transient pattern of proteins that facilitates efficient signal transduction through the creation of local, short-lived high-concentration signaling hubs that single molecules could not provide. In summary, weak noncovalent interactions yield HOTS, which provide a form of biomolecular assembly that is inherently transient and therefore well-suited to fulfill transient biological processes such as found, for instance, in signal transduction cascades.

HOTS underlie what we have termed dynamic connectivity (17). Dynamic connectivity differs from the notion of connectivity mediated by strongly interacting, static complexes, where protein A is tightly bound to protein B, B to C, etc. On the one hand, strong interactions, akin to hard wiring a circuit, would seem more efficient because most of the A proteins would be near B proteins instead of there being some small fraction of spatial coincidence. On the other hand, if this small fraction is sufficient to communicate the signal and yield a desired outcome, then dynamic connectivity offers several distinct advantages over hard wiring. First, as implied above, HOTS naturally exhibit lifetimes that are appropriate for rapid signaling. Second, signaling pathways ought to be switchable between on and off states. Weak interactions would permit rapid modification of pathway connectivity with, for instance, phosphorylation or ligand-induced conformational changes serving as regulatory switches. Third, multiple distinct signaling pathways typically coexist in a cell membrane and some components are shared. Dynamic connectivity naturally permits component sharing because only a fraction of the available molecules (and HOTS) are used to communicate a specific signal. The second and third advantages of dynamic connectivity (i.e., rapid on–off switching and component sharing) taken together describe circuits that could, in response to biochemical regulation of their cohesiveness, rapidly “rewire” themselves out of available components. At this early stage of research into HOTS these ideas require validation.

The arrows connecting signal transduction components in textbook figures do not convey mechanisms of interaction. At one extreme, localization of components might be unimportant for some pathways in which, for example, a second messenger is spread uniformly throughout the cell, while at the other extreme proteins at the two ends of the arrow might be permanently bound to each other to achieve highly focused localization. We suspect that life utilizes different levels of proximity among pathway components, mediated by a range of interactions from nonexistent to weak to strong. The existence of HOTS formed by membrane proteins and their quantitative explanation in terms of general principles of thermodynamics applied to weak but specific molecular interactions, suggest that dynamic connectivity might be ubiquitous. We wonder whether weak noncovalent interactions yield HOTS in three-dimensional compartments of cells, and whether HOTS more broadly underlie the connectivity of components in signaling pathways, metabolic pathways, and other forms of dynamic structure and function transcending single biomolecules.

## Data Availability

There are no data underlying this work.
